# MEMS Flow Sensors Based on Self-Heated aGe-Thermistors in a Wheatstone Bridge

**DOI:** 10.3390/s150510004

**Published:** 2015-04-28

**Authors:** Almir Talic, Samir Cerimovic, Roman Beigelbeck, Franz Kohl, Thilo Sauter, Franz Keplinger

**Affiliations:** 1 Center for Integrated Sensor Systems, Danube University Krems, Viktor-Kaplan Straße 2, A-2700 Wiener Neustadt, Austria; E-Mails: samir.cerimovic@donau-uni.ac.at (S.C.); roman.beigelbeck@donau-uni.ac.at (R.B.); kohl@iiss.at (F.K.); thilo.sauter@donau-uni.ac.at (T.S.); 2 Institute of Sensor and Actuator Systems, Vienna University of Technology, Gusshausstraße 27-29, A-1040 Vienna, Austria; E-Mail: franz.keplinger@tuwien.ac.at

**Keywords:** flow sensor, Finite Element Method, Wheatstone bridge configuration

## Abstract

A thermal flow transduction method combining the advantages of calorimetric and hot-film transduction principles is developed and analyzed by Finite Element Method (FEM) simulations and confirmed experimentally. The analyses include electrothermal feedback effects of current driven NTC thermistors. Four thin-film germanium thermistors acting simultaneously as heat sources and as temperature sensors are embedded in a micromachined silicon-nitride membrane. These devices form a self-heated Wheatstone bridge that is unbalanced by convective cooling. The voltage across the bridge and the total dissipated power are exploited as output quantities. The used thin-film thermistors feature an extremely high temperature sensitivity. Combined with properly designed resistance values, a power demand in sub-1mW range enables efficient gas-flow transduction, as confirmed by measurements. Two sensor configurations with different arrangements of the membrane thermistors were examined experimentally. Moreover, we investigated the influence of different layouts on the rise time, the sensitivity, and the usable flow range by means of two-dimensional finite element simulations. The simulation results are in reasonable agreement with corresponding measurement data confirming the basic assumptions and modeling approach.

## 1. Introduction

During the last decade, a growing demand for miniaturized flow sensors in industrial, automotive, medical, and domestic appliances has evolved. Some of the flow transduction principles proposed in the literature rely on micro-Prandtl tubes [1], resonating bridges [2], pyroelectric elements [3], thermopiles [4], and PTC or NTC thermistors [5,6,7]. For applications where local flow velocities are of interest, thermal flow transducers are beneficial since their performance can be improved by simply shrinking the relevant device dimensions. High sensitivity to flow, low cost, quick response, and low power consumption are the most important advantages of micromachined thermal flow sensors [8].

We used an advanced combination of two common transduction methods employed for thermal conversion of flow, namely calorimetric and hot-wire or hot-film flow transduction.

*Calorimetric flow sensors* typically consist of a miniaturized heat source in combination with spatially separated temperature sensors (e.g., thermistors), all embedded in thin membranes, cantilevers, or microbridges [5,6,9,10]. These thermistors sense local changes of the temperature at selected sites upstream and downstream of the heat source. In the case of a resting fluid, the generated temperature field is symmetrical with regard to the heat source due to the symmetric sensor layout. Any convective heat transfer along the sensor surface distorts the thermal symmetry inside the membrane. Approaching the heater, the fluid temperature increases in the flow direction. Hence, the cooling effect is more pronounced upstream of the heater and reduced—or even reversed—in the downstream area. The related downstream/upstream temperature difference is converted into an output voltage by a properly designed electronic circuit. The response of a calorimetric flow sensor depends basically on the geometrical parameters of the design, the operating power, the thermal properties of the involved materials, and on the thermal parameters of the fluid under investigation [7]. With increasing flow, the temperature difference increases up to a maximum and decreases afterwards since the entire sensor surface cools down. This maximum defines the feasible measuring range that can be exploited only if higher flow velocities are inhibited by the actual application. However, the measuring range can be extended towards higher flow rates if the dissipated electrical power is increased according to the cooling rate. Then the applied power can be used as a measure for mass flow in addition to the temperature difference signal that provides fluid velocity and flow direction information. The attainable flow measuring range and the sensitivity are strongly influenced by the distance between heater and temperature sensors and the membrane dimensions [3,4]. By means of multiple heating or sensing elements, the shape of the output characteristic, *i.e.*, the temperature difference *versus* flow rate can be designed [6].

*Hot-wire (hot-film)* flow sensors directly exploit the temperature of a miniaturized heat source exposed to the flow to be measured. A thin-film stripe of an electrical conductor featuring dimensions in the range of a few microns in the direction of the flow serves as heating and as temperature sensing element simultaneously. Compared to calorimetric conversion, the same power dissipation enables significantly higher excess temperature levels for flow conversion. Maintaining a constant excess temperature of the film, the required heating power can be utilized as sensor output, which is the preferable transduction mode at high flow rates. However, hot-wire flow conversion requires advanced electronic means for simultaneous power dissipation control on the one hand and precise estimation of temperature changes of the very same device on the other hand. Hot-film flow sensors *per se* do not allow for flow direction recognition.

Contrary to the simple calorimetric concept with an active thin-film heating resistor and symmetrically positioned temperature sensors [11,12], we study in this paper a thermal flow sensor that incorporates advantages of both the calorimetric and the hot-film transduction principle in a single device. This device is based on an array of four thin-film germanium thermistors embedded in a silicon membrane and connected to form a Wheatstone bridge. They simultaneously act as localized heat sources and temperature sensors and thus enable hot-film transduction. However, each thermistor resides in close vicinity to the other three devices resulting in a further, *i.e.*, calorimetric contribution to its excess temperature. The observed excess temperature of each thermistor depends on its own power dissipation, the heat supplied by the other thermistors, and the convective heat transfer. This interaction enables the extraction of the flow direction from temperature differences. A special feature of our design results from the negative temperature coefficient of resistivity (NTC) and the applied constant current supply of the Wheatstone bridge. In this case, convective cooling of the thermistors increases the Joule heat dissipated in these devices.

The benefits of the current approach are only partially offered by competitive transduction concepts. Extreme low-power and low temperature excess operation is hardly feasible with pure calorimetric transducers as the temperature of the heat source embedded in the membrane must always be much higher than that of the separated temperature sensors. The power consumption of the pure calorimetric conversion reported in [12] consumes for similar specifications a tenfold amount of power compared to the transducers of this contribution, although the same micromachining technology has been applied. A remarkable exception is the 2D flow sensor presented in [13]. This device offers calorimetric operation in the sub-mW region. However, the heater temperature elevation is still three times the excess temperature of the temperature sensors [14]. The thermopile-based calorimetric approach in [11] offers a low operational power (1mW) and low membrane excess temperature (~5 K). However, the reported transducer offers only 5% of the initial sensitivity of the current approach and uses the eightfold membrane area.

The thermal microsensor presented in [5] belongs to the rare applications of mixed-mode anemometric-calorimetric flow transduction. It offers superior response speed and sensitivity. The reported excess temperature of the hot wire structure amounts 60 K above ambient, which makes the device incompatible with delicate fluids applications.

We aim at flow measurements with minimum thermal distortion of the fluid to be measured in order to measure the motion of thermally sensible fluids. Low excess temperatures in the fluid are tantamount to low power consumption by the transduction mechanism. This feature is of great interest for distributed measurements by battery operated autarkic sensor devices. One major trend in modern thermal fluid sensing is the combination of alternative transduction principles in a single MEMS device. The authors of [15] successfully combined calorimetric and thermal time-of-flight conversion. Moreover, using advanced MEMS technologies, impressive multi-parameter measurements have been achieved through a combination of thermal and Coriolis flow transducers with a pressure sensor [16]. In contrast, the current work focuses on the establishment of design guidelines for a single mixed-mode thermal flow transducer. The presented approach excels with a combination of high sensitivity, low power consumption, and low excess temperatures required for operation.

## 2. Flow Sensor Design and Fabrication

Thin-film thermistors based on semiconducting films are favorable temperature sensors for thermal flow sensing. Their advantages are given by the desirable combination of small size, variability of shape, and high temperature resolution. Furthermore, they offer high signal levels at low operating currents based on a high specific resistivity. Thermistors do not need cold junction compensation because their resistance is a function of the absolute temperature. Semiconductor thermistors are high resistivity devices, which allows precise resistance measurements based on power dissipations of the order of 1 µW. For the current application, thermistor devices are needed that endure power dissipations on the order of 100 µW in spite of the thermal decoupling from massive heat sinks by being incorporated in a thin membrane.

**Figure 1:**
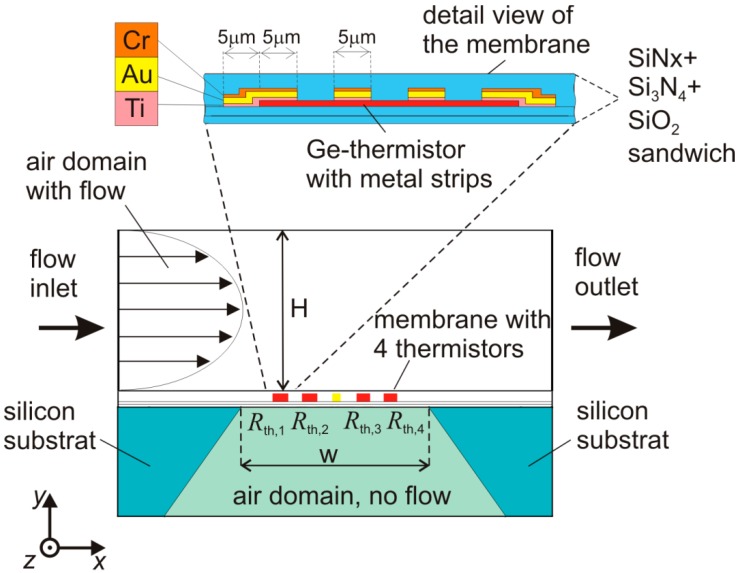
Schematic cross section of the flow sensor. Membrane thermistors (*R*_th,1_ to *R*_th,4_) are used as thermal actuators and temperature transducers simultaneously. The inset details the thin-film construction of a single thermistor comprising interdigitated electric contacts.

Figure 1 illustrates a cross-sectional view of the investigated flow sensors. Four thermistors are symmetrically arranged relative to a central chromium resistor that can be used to study conventional calorimetric conversion. Amorphous germanium excels with high resistivity values of about 5 Ωm at room temperature. Moreover, it offers a high temperature coefficient of resistivity, *TCR* = *R*^−1^·*dR*/*dT*, of about −0.02/°C, where *R* denotes the electrical resistance of the thermistor and *T* the temperature. The high *TCR* of amorphous germanium, in combination with a low 1/f noise level, enables thermistor designs featuring a temperature resolution better than 0.3 mK/√Hz [17]. Measurements of the temperature dependence of the thermistor resistance between 77 and 330 K revealed that the electrical conductivity of amorphous germanium is governed by a variable range hopping process [18]. At room temperature, the *TCR* varies only slightly with the temperature, which eases appreciably the burden for compensation of ambient temperature variations. Each thermistor features a resistance of about 80 kΩ at room temperature.

**Figure 2:**
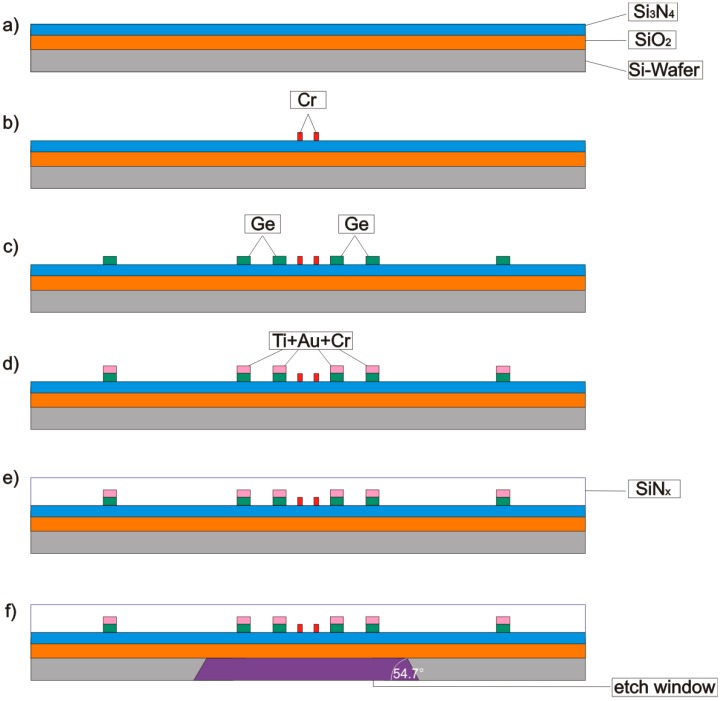
Sequence of main fabrication steps. Thicknesses of individual layers are not scaled.

The flow sensor chips were fabricated according to the process flow illustrated in Figure 2, where cleaning and surface activation steps have been omitted:

(a) The starting material is a 350 µm thick, <100> *p*-type, double side polished silicon wafer with a 100 mm diameter. The wafer was coated by the vendor with thermally grown silicon dioxide (SiO_2_) and LPCVD silicon nitride (Si_3_N_4_) featuring a thickness of 250 nm and 70 nm, respectively.
(b) The sensor fabrication starts with a photolithographic process and a high-vacuum vapor-deposition of a 260 nm thick layer of germanium using an electron beam evaporator. Subsequently, thermistor areas are structured using the lift-off technique.
(c) Next, the heater shapes are created by a second lithography, evaporation of a 130 nm thick chromium layer, and lift-off patterning. These structures are intended for comparison of the transduction studied in this contribution with conventional calorimetric transduction treated elsewhere [19].
(d) Photolithographic lift-off techniques are also employed to obtain interdigitated electrodes for the thermistors as well as connection leads from the heater and the thermistors to the bonding pads. A titanium-gold-chromium sandwich layer featuring thicknesses of 70-130-50 nm is used for this purpose. The complete thermistors structure measures 45 µm along the intended direction of flow by 600 µm in the perpendicular direction.
(e) Afterwards, sensor surface and sensor elements are covered with a silicon nitride layer. A low stress silicon nitride (SiN_X_) protective film of a thickness of about 1250 nm is applied by means of a low temperature plasma enhanced chemical vapor deposition (PECVD) process (under 200 °C). The deposited PECVD silicon nitride exhibits a very low thermal conductivity of about 1.2 WK^−1^m^−1^ as compared to 150 WK^−1^m^−1^ for silicon [20]. The minimum thickness of the SiN_X_ layer of the available sensor technology is determined (i) by mechanical stability considerations and (ii) by sufficient step coverage of the surface discontinuities introduced by the thin-film structures. The resulting thickness of the PECVD SiN_X_ layer causes more than the desired heat flow within the membrane. The high strength of thicker membranes helps them to withstand mechanical loads, e.g., during the subsequent through-wafer etching process, or for transient flow studies based on tube shock waves. Using stress-optimized deposition methods for SiN_X_ facilitates further reduction of the diaphragm thickness, yielding higher flow sensitivities and faster response.
(f) In order to obtain access to the bond pads, the protective SiN_X_ film is selectively removed from the front-side using photolithography and reactive ion etching (RIE). Finally, the chromium is removed from the bond pad metallization by means of a selective wet-etching process. An annealing process is applied to assure long term stability of resistivity and NTC of the amorphous germanium layer, comprising three steps at 100 °C, 130 °C and 150 °C for a few hours each. Next, square apertures are etched into the wafer backside coating by means of photolithography and reactive ion etching (RIE). The membrane is then manufactured using a 30% KOH based anisotropic wet etching process (at 75 °C) to remove the bulk silicon from the backside of the wafer. The front side of the wafer is protected from the etchant using a custom-made holder. The membrane consisting of silicon dioxide and both silicon nitride layers features an overall thickness of about 1.57 µm. Finally, dicing delivers individual chips of size 3 × 6 × 0.35 mm^3^.

**Figure 3:**
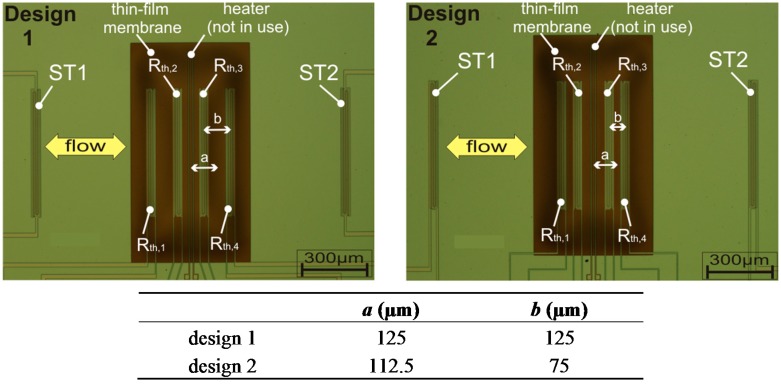
Photomicrograph of the flow sensor chip (design embodiment 1 and 2). Membrane size: 0.5 × 1 mm^2^. The table shows design variations (distances *a*, *b*).

Two fabricated sensor configurations are depicted in Figure 3. The investigated designs exhibit different arrangements of the membrane thermistors (discriminated by the indicated distances *a* and *b*). The additional substrate thermistors estimate the chip temperature that is close to the ambient and fluid temperatures (ST1 and ST2). The central chromium resistor can be used as an additional heater, but for the current study we utilized only the self-heating of the membrane thermistors as the heat source. Activating this thin-film metallic heater, the sensor offers two additional operating modes as a common calorimetric flow sensor [19]. In the first additional mode, the device could also be used with the central heat source and the pair of inner, or alternatively, outer thermistors as temperature sensors. In the second mode, all membrane thermistors are connected to form a Wheatstone bridge for the signal readout, and the chromium resistor remains as the heat source.

## 3. Simulation Setup

Finite element (FE) simulations are an efficient tool to determine the temperature distribution within the sensor membrane. The conjugated heat transfer can be simulated on standard PCs with moderate effort as long as one employs a two-dimensional (2D) approximation.

Using FE computations [21], we simulated the application of nitrogen gas flow and investigated possible design improvements. The time-dependent FE analysis is based on a 2D model corresponding to the schematic cross section shown in Figure 1. The assumed flow channel height corresponds to the intended experimental arrangement. Based on a reduced heat transfer equation, the employed heat transport model features conduction and convection, whereas radiative heat transfer effects as well as the temperature dependencies of the viscosity, the density, the heat capacity, and thermal conductivity of the fluid have been neglected.

(1) $$\rho C_{\text{p}}\frac{\partial T}{\partial t}+\nabla \cdot (-k\nabla T+\rho C_{\text{p}}Tu)=Q$$

where *T*, *ρ*, *k*, and *C*_p_ denote the temperature, the density, the thermal conductivity, and the heat capacity of the medium, respectively. *Q* denotes the density of the supplied heat source and **u** represents the local velocity. The sensor model comprises gas domains below and above the sensor’s membrane. Convective heat transfer is only considered in the upper one. In flow direction, the model of the sensor membrane equals the physical dimension of 0.5 mm, whereas the Si chip dimension is reduced from 3 to 1.4 mm to keep the demand for computational power low. Perpendicular to the cross section of Figure 1, the model extension is assumed to be infinite. The flow is considered to be laminar and parallel to the sensor membrane and the fluid velocity in *x*-direction is imposed as a parabolic function with respect to the *y*-coordinate, implying the non-slip boundary condition at the bottom and top of the flow channel.

The boundary condition at the outlet of the flow compartment is implemented as convective flux; the remaining parts of the model circumference are kept at ambient temperature (*T*_amb_ = 21 °C).

Perpendicular to the flow velocity, the extension of the thin-film thermistors and the flow channel measure 0.6 mm and 1.2 mm, respectively. As these values are comparable with the channel height, approximate corrections have to be introduced in the FE model to account for 3D effects.

First, the average velocity profile above the thin film thermistors has to be computed from the mean flow velocity in the flow channel. Assuming a fully developed Poiseuille flow, the velocity profile in the rectangular flow channel is given by:

(2) $$v_{x,\text{3D}}\left(y,z\right)=v_{\max}\;\left(1-{\left(\frac{\left(y-H/2\right)}{H/2}\right)}^{2}\right)\;\left(1-{\left(\frac{\left(z-W/2\right)}{W/2}\right)}^{2}\right)$$

where *H* denotes the channel height, *W* the channel width, *v*_max_ the peak velocity, and *y*, *z* are coordinates of the cross section measured from a corner of the rectangle. The peak flow velocity in the center of the flow channel amounts to 2.25 ∙ *v*_mean_, where *v*_mean_ denotes the average flow velocity of the channel cross section. To compute the effective flow profile of the 2D model, the *z*-dependence of *v_x_*_,3D_ has to be appropriately considered by taking the average flow profile above the thermistors, which measures only 600 µm = *W*/2 in *z*-direction:

(3) $$v_{\text{2D}}\left(y\right)=\left(1-\frac{{\left(y-H/2\right)}^{2}}{{\left(H/2\right)}^{2}}\right)\;v_{\max}\;\frac{2}{W}\;\int_{W/4}^{3W/4}\left(1-\frac{{\left(z-W/2\right)}^{2}}{{\left(W/2\right)}^{2}}\right)\;dz=2.0625\;v_{\text{mean}}\;\left(1-\frac{{\left(y-H/2\right)}^{2}}{{\left(H/2\right)}^{2}}\right)$$

Second, the thermal conductivities and mass densities of the membrane constituents have to be appropriately scaled since the excess temperature field in the membrane extends somewhat beyond the *z*-dimension of the thermistors. We found empirically that a scaling factor of *C*_2D_ = 1.35 referring to the ratio of simulation parameters/physical parameters of membrane materials enables a good agreement of measured characteristic and simulation results.

**Figure 4:**
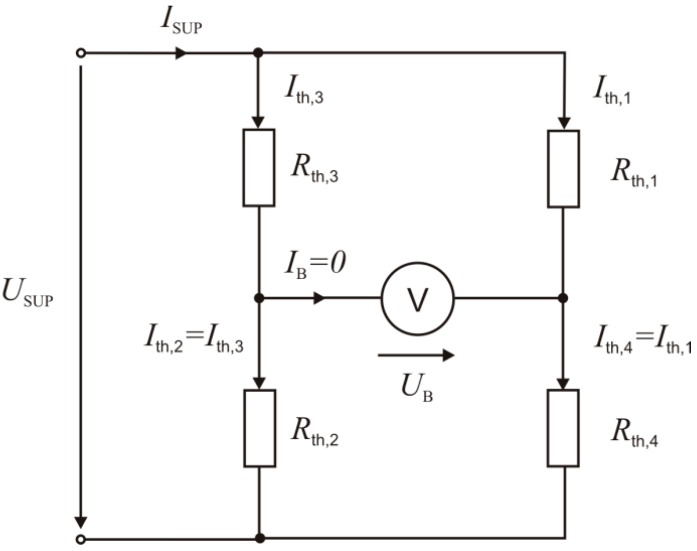
Wheatstone bridge featuring four membrane thermistors. The bridge is supplied by a constant current *I*_SUP_ = 70 µA. Both voltages *U*_B_ and *U*_SUP_ are flow dependent.

The electrical resistance of the thermistors is modeled as:

(4) $$R_{\text{th,}i}(\vartheta )=R_{\text{th,0}}\;e^{\alpha \vartheta_{i}},\;i=1\cdots 4$$

where *α* = −0.02/°C is the measured temperature coefficient of resistivity and *R*_th,0_ ≈ 125 kΩ is the thermistor resistance at 0 °C. The thermistor temperatures *ϑ_i_* were calculated through a subdomain integration of the variable *T* over each thermistor area. The thermistors are suitably connected to form a Wheatstone bridge (according to Figure 4), which enables optimal flow sensitivity as well as the bidirectional characteristic of the output signal. Due to self-heating, these thermistors operate simultaneously as heat sources as well as temperature sensors. To avoid thermal destruction of the NTC thermistors, the bridge was supplied by a constant electric current *I*_SUP_ = 70 µA, ensuring that the thermistor voltages do not exceed the maximum value of 6 V.

The density of the dissipated power in the individual thermistor equals

(5) $$Q_{i}=\frac{I_{\text{th,}i}^{2}\;R_{\text{th,}i}(\vartheta_{i})}{V},\;i=1...4$$

and depends indirectly on the device temperature *ϑ_i_* owing to the thermistor effect. *V* specifies the thermistor volume and *I*_th,i_ is the thermistor current:

(6) $$I_{\text{th,2}}=I_{\text{th,3}}=I_{\text{SUP}}\frac{R_{\text{th,1}}\left(\vartheta_{1}\right)+R_{\text{th,4}}\left(\vartheta_{4}\right)}{R_{\text{th,1}}\left(\vartheta_{1}\right)+R_{\text{th,2}}\left(\vartheta_{2}\right)+R_{\text{th,3}}\left(\vartheta_{3}\right)+R_{\text{th,4}}\left(\vartheta_{4}\right)}$$

(7) $$I_{\text{th,1}}=I_{\text{th,4}}=I_{\text{SUP}}\frac{R_{\text{th,2}}\left(\vartheta_{2}\right)+R_{\text{th,3}}\left(\vartheta_{3}\right)}{R_{\text{th,1}}\left(\vartheta_{1}\right)+R_{\text{th,2}}\left(\vartheta_{2}\right)+R_{\text{th,3}}\left(\vartheta_{3}\right)+R_{\text{th,4}}\left(\vartheta_{4}\right)}$$

**Figure 5:**
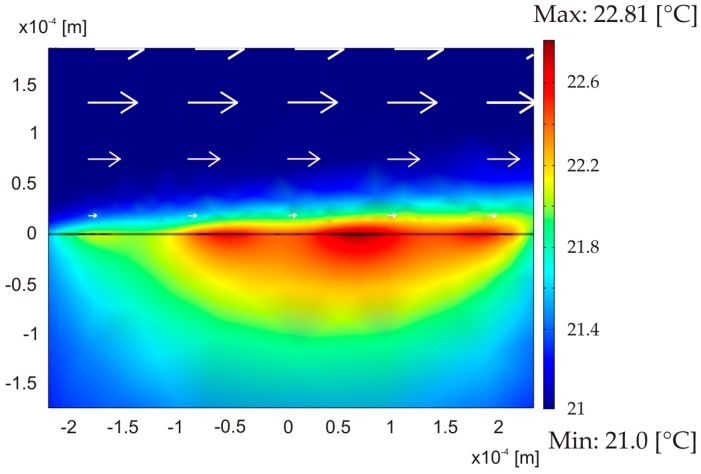
Simulated temperature distribution at the symmetry plane of the sensor for an average flow velocity of *v*_mean_ = 20 m/s and a supply current of *I*_SUP_ = 70 µA (design 1). Boundary temperatures were set at ambient temperature *T*_amb_ = 21 °C.

Each thermistor temperature *ϑ_i_* is determined by the conjugated heat transfer on the one hand and the power dissipation *Q_i_* on the other hand, which in turn depends on the temperature. Starting from the initial temperature, the values *R*_th,*i*_, *I*_th,*i*_, and hence *Q_i_* are computed. The first value of *Q_i_* is imposed as heat supply to the model and the thermistor temperatures *ϑ_i_* are calculated for a prescribed mean velocity through subdomain integrals of the simulated temperature for each thermistor area. In subsequent computational steps, *Q_i_* is updated and the thermistor temperatures are estimated anew until convergence is reached. Then the bridge voltage and the total dissipated power are evaluated from the thermistor’s resistances. Figure 5 visualizes a typical example of a simulated 2D temperature field in the membrane region for a flow velocity of 20 m/s and a total dissipated power of about 380 µW (design 1).

**Figure 6:**
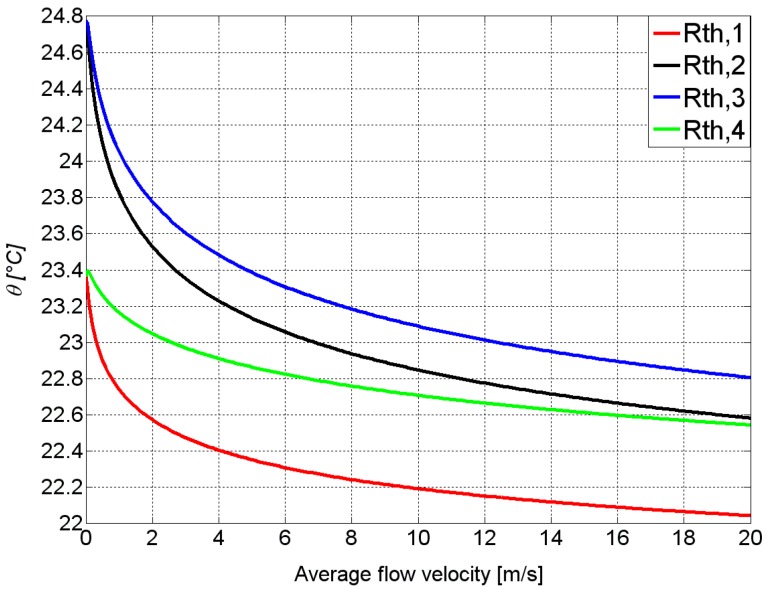
Simulated dependence of the thermistor temperatures (for design 1) on the flow velocity. The temperature of the incoming fluid and the ambient temperature were both set to 21 °C. The thermistors Th_2_ and Th_3_ are closer to the membrane center (black and blue lines) and therefore show a higher initial temperature compared to the outmost thermistors (green and red lines). The efficiency of convective cooling decreases from Th_1_ to Th_4_, hence the characteristics of Th_2_ and Th_4_ intersect. Moreover, at very low flow velocities, the temperatures of the downstream thermistors Th_3_ and Th_4_ exhibit a slight elevation of their temperatures over their respective values at zero flow.

Figure 6 shows the simulated dependencies of the thermistor temperatures *ϑ*_i_ on the average flow velocity for design 1. The thermistors in the silicon nitride membrane act as symmetrically embedded heat sources. Hence the temperature profile inside the membrane is symmetrical in case of zero flow. Therefore, the outer thermistors *R*_th,1_ and *R*_th,4_ exhibit the same temperature. The inner thermistors *R*_th,2_ and *R*_th,3_ behave in the same way, but with a higher excess temperature. The fluid flow passing by the membrane surface deforms the temperature profile inside the membrane, which is converted into flow magnitude and direction signals by means of the thermistor bridge. Symmetrically located thermistors exhibit different flow dependencies. Upstream positioned thermistors are cooled down very efficiently compared with their downstream counterparts, which may exhibit a slight temperature increase for very low flow velocities. This asymmetric behavior indicates a significant overlap of their temperature fields, which is mandatory for a calorimetric response to a fluid flowing across the membrane.

The voltages across the bridge *U*_B_ as well as that at the bridge supply terminals *U*_SUP_ depend on the thermistor resistances and hence on the flow velocity:

(8) $$U_{\text{B}}=I_{\text{SUP}}\frac{R_{\text{th,1}}R_{\text{th,2}}-R_{\text{th,3}}R_{\text{th,4}}}{R_{\text{th,1}}+R_{\text{th,2}}+R_{\text{th,3}}+R_{\text{th,4}}}$$

(9) $$U_{\text{SUP}}=I_{\text{SUP}}\frac{\left(R_{\text{th,2}}+R_{\text{th,3}})(R_{\text{th,1}}+R_{\text{th,4}}\right)}{R_{\text{th,1}}+R_{\text{th,2}}+R_{\text{th,3}}+R_{\text{th,4}}}$$

The flow dependence of these output variables is obtained from several simulation runs done for appropriately selected values of *v*_mean_. Adequate locations of the thermistors on the membrane resulting in initial flow sensitivity or a wide velocity range of monotonous transduction can be found from a set of such simulations.

## 4. Measurement Setup

**Figure 7:**
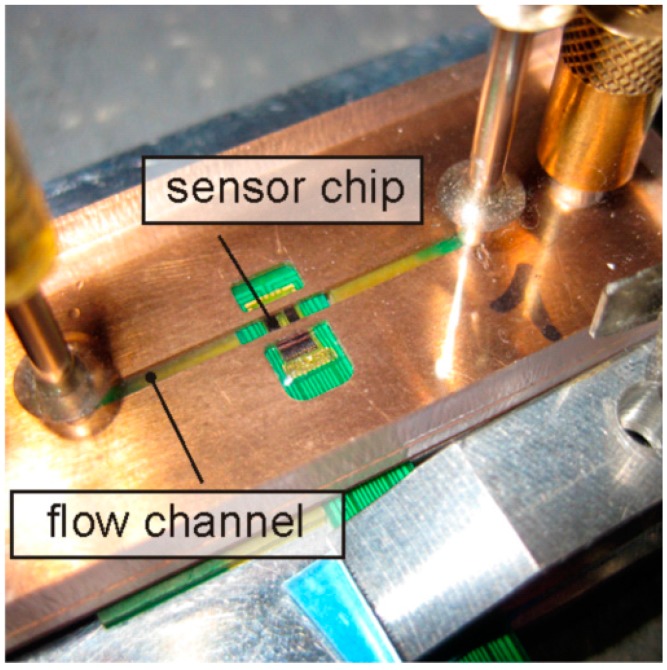
Sensor chip incorporated in a printed circuit board (PCB) and then flush mounted into the wall of miniaturized flow channel with cross-sectional dimensions 1.2 × 0.5 mm^2^.

To study the stationary flow transduction, the sensor chip was accommodated in a milled recess of a 1.0 mm thick printed circuit board (PCB) flush with its surface. This PCB constitutes the bottom wall of a rectangular flow duct used for sensor characterization, featuring 1.2 mm width, 0.5 mm height, and 25 mm length (Figure 7).

The side walls of the duct were machined from copper plate whereas transparent Lucite was used as a top cover. Mean flow velocities of N_2_ of up to 27.5 m/s were established by two standard mass flow controllers featuring ranges of 100 and 2000 cm^3^/min, respectively. This arrangement is best suited for comparison of simulations and experiment since it establishes a well-defined plane Poiseuille velocity field for a wide range of flow rates.

Digital multimeters (model Agilent 34410A) were used to record *U*_B_ as well as the bridge supply voltage *U*_SUP_. The fluid and chip temperatures were recorded by using four-wire PT100 temperature sensors placed in the flow tubing and at the aluminum base plate of the setup, respectively. Current-driven NTC thermistors show increasing power dissipation with decreasing device temperature. Hence, a slight negative electro-thermal feedback is established by the thermistors themselves with respect to convective cooling. For example, thermistor *R*_th,2_ gains power dissipation at the cost of *R*_th,3_. Negative feedback leads to a wider measuring range on the one hand but reduced voltage signals on the other hand. Moreover, constant current-driven NTC thermistors are protected against thermal destruction, which is a major threat of constant voltage operation that would cause positive electrothermal feedback in the case of raising ambient temperature [22]. Although the Wheatstone bridge is current supplied, the individual NTC thermistor in this case, devices are not. According to the chosen bridge configuration, the splitting of the total supply current depends on the ratio of the total resistances of inner and outer thermistor pairs.

In principle, NTC thermistors may also be operated by a voltage source with a suitable series resistor, although the voltage at the thermistor terminals may decrease with increasing current in case of massive self-heating. To compensate for the negative resistance of the NTC thermistors, the value of the series resistor must be high enough to maintain thermal stability. Self-heating, however, depends on the change of temperature with dissipated power, *i.e.*, on the thermal parameters of the system. In the case of flow sensors, these parameters are subjected to significant change due to convective heat transfer, which impedes the design of the series resistance for safe operation.

## 5. Results

**Figure 8:**
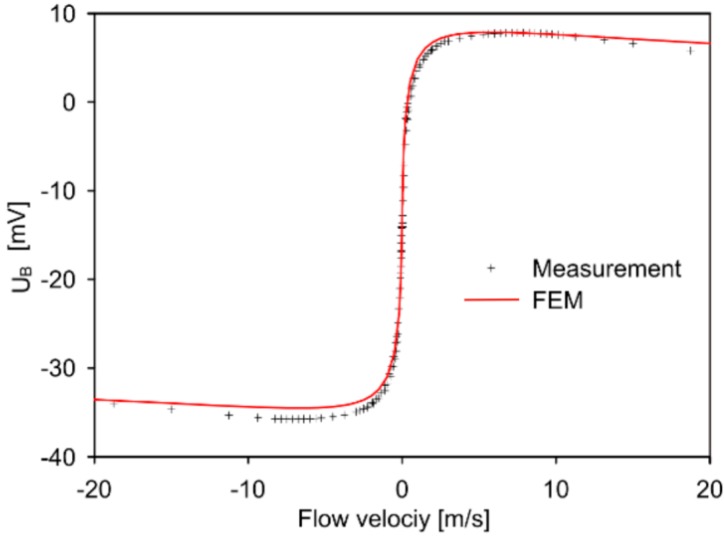
Measured and simulated dependence of *U*_B_ on the mean flow velocity for design 1 at ambient temperature of 22.2 °C. The main parameters of the measured characteristic, *i.e.*, the extrema of *U*_B_ appearing at the velocities *v*_peak+_ and *v*_peak−_ as well as the slope *s* of the characteristic at zero flow are listed in Table 1. Two flow controllers of 100 cm^3^/min and 2000 cm^3^/min capacity were alternately used to cover the maximum flow range.

Figure 8 displays the measured and simulated transduction characteristics of design 1 for mean flow velocities up to ±20 m/s. The zero-flow offset of *U*_B,off_ = −14.2 mV results from scattered thermistor resistances as confirmed by FE analyses. The simulated characteristic is based on equal fluid and chip temperatures of 22.2 °C and corresponding thermistor resistance values. The latter were derived from measured resistance values, which were sequentially taken at zero flow and an ambient temperature of 21.5 °C using an Agilent 34410A digital multimeter. The measured values were 81.440, 80.660, 80.774 and 82.150 Ω for *R*_th,1_, *R*_th,2_, *R*_th,3_, and *R*_th,4_, respectively. The values used for the simulation are corrected only for the different ambient temperatures. The corrected values deviate from the actual values due to the significant self-heating effect: For transducer operation, all thermistors are heated simultaneously and the operating current in the branches of the Wheatstone bridge differs from the measuring current applied by the multimeter. Hence, a small systematic deviation between the resistance data applied for the simulations and the thermistor’s resistances constituting the operating bridge occurs. Further deviation of measured and simulated *U*_B_ characteristics can be attributed to the approximations inherent to the 2D FE model. Nevertheless, a reasonable agreement of simulated and measured characteristics is obtained.

Due to a zero-flow slope of *s* ≈ 100 mV/(m/s), the bridge voltage *U*_B_ is the preferred output quantity for low flow velocities. However, the non-monotonous characteristic limits the applicability of this conversion method to a few m/s only. For flow transduction, the inverse characteristic of Figure 8 must be exploited. If the velocity in the duct might exceed *v*_peak_ significantly, the interpretation of the flow signal is no more unique, even for velocities |*v*_mean_| < *v*_peak±_. Moreover, because of the flat maximum, the practical range for duct flow measurements will be reduced to |*v*_mean_| < *v*_peak±_ too. An extension of the measuring range is feasible, however, by proper control of the heating current as in the case of constant excess temperature operation. Due to the highly efficient flow conversion, the heat input generated by the thermistor current may be reduced at low flow velocities in most applications.

The shape of the measured transduction characteristic is very common for calorimetric flow transducers operated with (nearly) constant power. The sign of the slope of *U*_B_ (*v*_mean_) changes from positive to negative at mean velocities *v*_peak+_ and *v*_peak−_. Outside this range, the signal decreases on both sides due to heavy convective cooling of the entire membrane [6].

Both transduction characteristics of Figure 8 deviate from perfect anti-symmetry for two reasons: 

*First*, *electrical asymmetry causes* *U*_B_ = *U*_B,off_ ≠ 0 at zero flow in the case of electrical asymmetry (*R*_th,1_*R*_th,2_ ≠ *R*_th,3_*R*_th,4_). This bridge detuning is typical for the implemented devices, as no resistance trimming technology was available. A typical resistance mismatch of about 2% between a single thermistor and all others, for instance, leads to a pure electrical bridge detuning *U*_B,off_ of about 25 mV at zero flow. The sign of the offset depends on both the sign of the resistance deviation and on the actual location of the outlier upstream or downstream of the membrane center. There is only a slight dependence of the offset magnitude on the distance of the mismatch from the membrane center. Resistance mismatch leads to an asymmetric distribution of the dissipated heat, which causes a thermal contribution to the transducer offset. In the case of a current supply, thermistors exhibiting larger resistances dissipate more power than low resistance devices. As more heat dissipation gives rise to higher device temperatures, the resistance deviations of NTC thermistors are somewhat reduced by the negative electro-thermal feedback effect. Thus the effect of the electrical mismatch is reduced.

*Second*, *thermal asymmetry* also affects the shape of the flow transduction characteristic due to the calorimetric component of the flow transduction. The actual self-heating of each thermistor depends on the local conductive and convective heat transfer, on its locally dissipated power, and, to a lesser extent, on the power dissipation of all other thermistors. In the case of more power dissipation at the upstream thermistor, there are two enhancements to the bridge detuning by convection. First, there is improved convective cooling of the upstream thermistors due to their larger self-heating, which is accompanied by the opposite effect in the downstream region. Second, enhanced heat transfer from the upstream thermistors to the fluid increases the fluid temperature downstream of these heat sources and reduces the heat transfer to the fluid in the downstream region. In the case of more heat dissipation in the downstream region, there is no convective heat transfer to the upstream thermistors at all.

**Figure 9:**
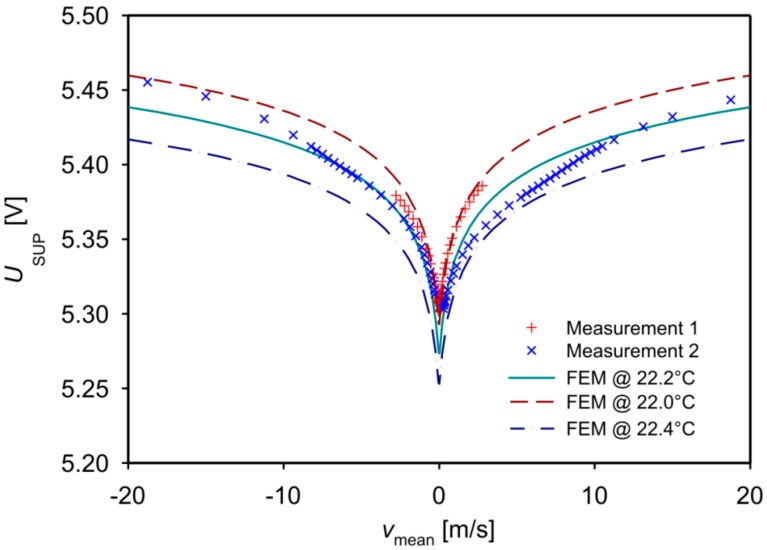
Measured and simulated dependence of *U*_SUP_ on the mean flow velocity in the duct (design 1). Flow controllers of 100 cm^3^/min (+) and 2000 cm^3^/min (×) capacity were used alternately to establish the flow. The simulated characteristics assume equal values for the fluid and chip temperature.

Figure 9 shows the measured dependence of the bridge supply voltage on the mean flow velocity, together with simulation results. The experimental data are not corrected for variations of ambient and fluid temperatures or for nonlinearity of the employed flow controller. In contrast to *U*_B_, the characteristic of *U*_SUP_ exhibits a monotonous flow dependence and sufficient sensitivity to be used as a complementary output signal.

**Figure 10:**
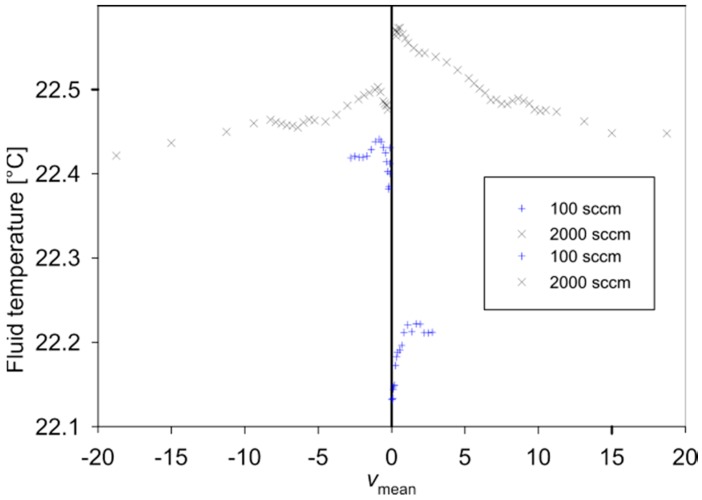
Measured fluid temperature during the measurement runs for the two flow transduction characteristics.

The relative deviation between individual measured characteristics of up to 0.6% results mainly from slight variations of the fluid temperature during the measurement runs. The fluid temperature records shown in Figure 10 were taken about 100 mm before the entrance of the measuring setup of Figure 7 using a thin-film Pt100 sensor inserted in the gas supply tube.

The total dissipated power *P*_SUP_ = *I*_SUP_∙*U*_SUP_ amounts to approximately 380 µW for typical thermistor resistances of 80 kΩ at ambient temperature. If one uses the *U*_SUP_ signal in conjunction with the *U*_B_ characteristic, an unambiguous interpretation of the *U*_B_ signal is ensured for all velocities.

We investigated two transducer configurations featuring different arrangements of the membrane thermistors (design embodiment 1 and 2 with different *a* and *b* shown in Table 1) experimentally. The simulated results are gathered together with experimental data in Table 1. The simulations include the actual mismatch of the membrane thermistor resistances.

**Table 1:** Sensor specifications for designs 1 and 2.

	Design 1				Design 2		
*a/b* (µm)	125/125				112.5/75		
	Measurement		Simulation		Measurement	Simulation	
*U*_B,off_ (mV)	−14.1		−12.95		−7.0	−6.23	
*s* (mV∙s/m)	62		82.5		93.7	108	
*U*_B,peak_ (mV)	7.8	−35.8	8	−34.7	17.58	18.9	−31.8
*U*_B,peak_ − *U*_B,off_ (mV)	21.9	−21.7	20.95	−21.75	24.58	25.13.	−25.75
v_peak+_, v_peak−_ (m/s)	7.9	−7.1	6.0	−6.5	6.75	5	−5.5

According to Table 1, there is a reasonable agreement between measured and simulated parameters with the exception that the simulated *v*_peak_ values deviate markedly from measured data. However, both the simulated and the measured *U*_B_ (*v*_mean_) characteristics show a rather flat maximum, which makes this comparison vulnerable to errors (see Figure 8). The difference between simulation and measurement probably results from minor differences between the experimental conditions and simulation parameters. The simulated characteristic of the offset-compensated bridge detuning voltage, *U*_B,peak_ − *U*_B,off_, exhibits deviations from pure antisymmetry, which reflects the influence of the thermal asymmetry introduced by non-ideal thermistor resistance values.

A positive value of *U*_B,off_ tends to increase the simulated values of *v*_peak+_ and decrease the magnitude of *v*_peak−_, whereas a negative offset causes opposite changes. However, this finding is incompatible with the measured data of design 1. Figure 10 points to one main reason for this inconsistency: Successive measurement runs of *U*_SUP_ over *v*_mean_ show considerable deviations that correlate with fluid temperature recordings shown in Figure 10 that were taken simultaneously. According to Equations (4) and (9), *U*_SUP_ increases with decreasing fluid and ambient temperatures if the Wheatstone bridge is composed of NTC resistors. A similar effect is expected from Equations (4) and (8) for the bridge detuning voltage and contributes to the observed deviations between measurements and simulations, e.g., *v*_max+_ and *v*_max−_ listed in Table 1. Nevertheless, the reasonable agreement between FEM simulation data and measurement results for the flow transduction characteristic confirms the validity of the FEM-based design study.

A main disadvantage of the sensor results from interference by chip and fluid temperature variations. The variation of temperatures does not affect the directional characteristics but influences the shape of the sensor characteristic. To reduce the temperature dependence of the measurement to a minimum, the fluid temperature could be measured by additional thermistors ST1 and ST2 and at the flow inlet. The temperature effect can then be eliminated using an appropriate correction model. Our sensor generates chip excess temperatures of a fraction of one degree and therefore all measured values are related to ambient temperature.

Calibrated thermal transducers are capable for operation in filtered gases of constant composition. However, care must be taken to avoid condensation of fluid constituents at the transducer surface, especially in the case of low excess temperature transducers. The static pressure range for useful gas flow transduction may extend from fractions of an atmosphere to the MPa region as long as a moderate pressure dependence of the thermal properties of the fluid is ensured. Dynamic pressure variations are permissible only if the setup ensures zero differential pressure at the transducer membrane at all times.

## 6. FE Analyses of Layout Variations

Based on the highly efficient conversion of small flow velocities and the moderate flow velocity limit introduced by the slope reversal, proper transducer design rules governing these characteristic parameters are of great interest.

The influence of variations of thermistor positions are computed for a sensor exhibiting a thermistor extension of 45 µm and a membrane width of *w* = 524 µm, both measured in the direction of flow. The distances *a* and *b* (Figure 3) between the thermistors were varied systematically. All simulations assume perfect thermistor matching and equal temperatures of chip and incoming fluid.

**Figure 11:**
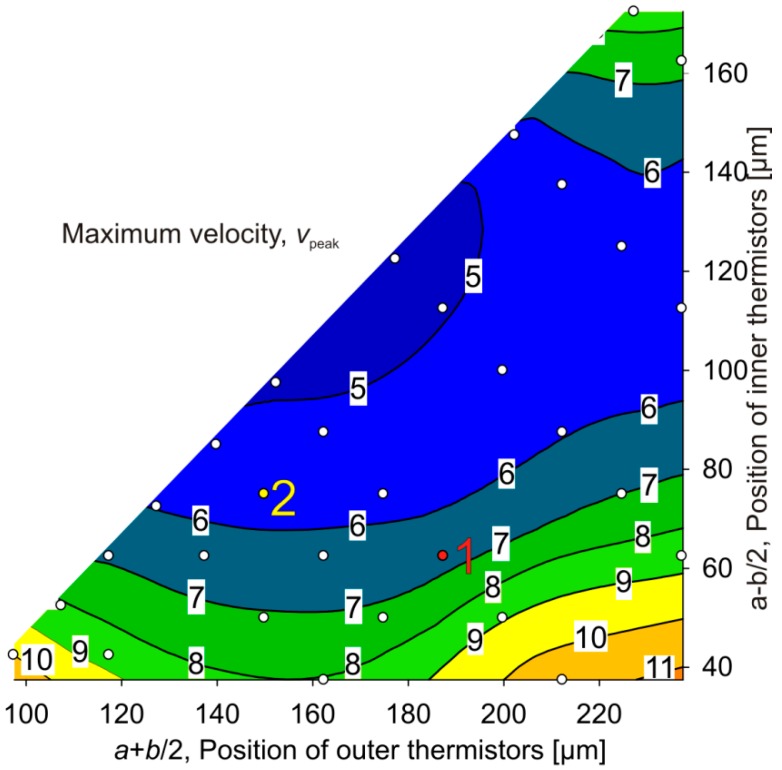
Contour plot of the simulated velocity *v*_peak_ [m/s], which marks the boundary of the monotonous transduction characteristic. The available region for possible designs is plotted using the position of inner and outer thermistors as design parameters. The designs of implemented devices are indicated by respective numbers (designs 1 and 2).

Figure 11 shows a contour plot of simulation results for the range of monotonous transduction *v*_peak_ as a function of the position of the inner and outer thermistors. The locations of Th2 or Th3 (vertical axis) and Th1 or Th4 (horizontal axis) are measured from the middle of the membrane and indicated as open circles. The contour plot is based on an interpolation of 30 simulated design variants and also illustrates the available design space for thermistor positioning on the membrane area (Figure 3).

Figure 11 reveals three boundary maxima of *v*_peak_. For large values of *v*_peak_, the inner thermistors should be placed either together with the outer thermistors as close as possible at the membrane border (top corner of the design space), or as close as possible to the membrane center (bottom boundary of the design space). The latter approach offers two possibilities for the outer thermistor positions which deliver good *v*_peak_ performance, *i.e.*, either close to inner thermistors or close to the membrane edge. Designs at the lower border have in common that upstream Th2 and downstream Th3 are as closely spaced as feasible. Therefore, convective heat transfer has to compete with the tight thermal coupling of these thermistors by heat conduction: Assuming cold gas flow from left to right, the temperature of Th2 decreases more than the temperature of Th3, which enhances conductive heat transfer from Th3 towards Th2, *i.e.*, in the opposite direction as convective heat transfer. Hence the temperature difference between Th3 and Th2 sites induced by convection is lower the stronger their thermal coupling by heat conduction through the membrane as well as through the top and bottom of gas compartments. For the closest approach of Th2 and Th3, the position of the outer pair of thermistors, Th1 and Th4, causes a saddle minimum at *a* + *b*/2 ≈ *w*/4. This membrane region is characterized by weak thermal conduction both to the heat sink and the neighboring thermistor and hence high sensitivity against convective heat transfer. There Th1 and Th4 contribute strongly to the bridge detuning at low flow velocities, but their temperature difference peaks at rather low flow velocities due to efficient convective cooling.

The top corner region of enhanced *v*_peak_ (Figure 11) is characterized by closely spaced thermistors pairs featuring positions near the membrane border. Here *v*_peak_ is relatively high since convective heat transfer competes with the enhanced heat conduction to the nearby silicon frame.

**Figure 12:**
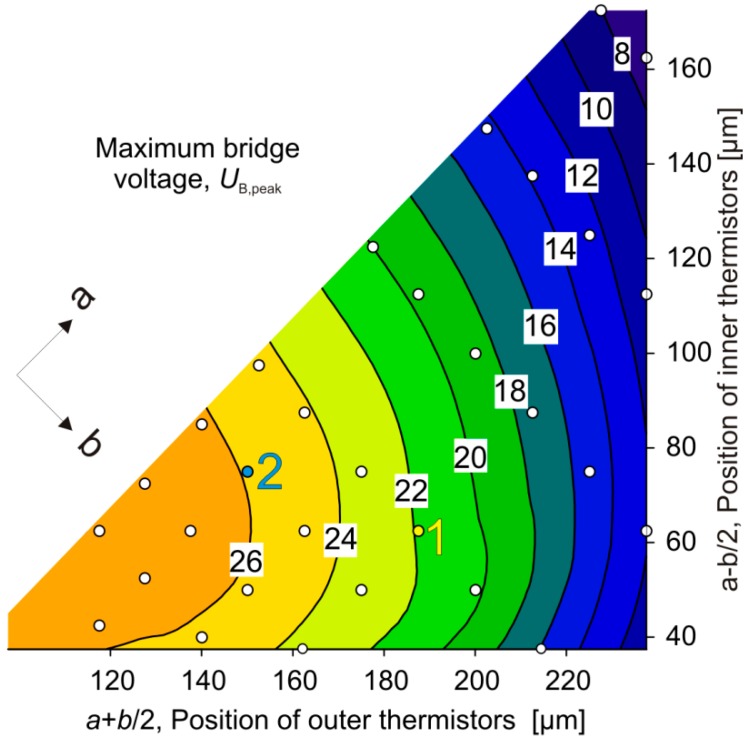
Contour plot of the maximum bridge detuning voltage simulated as a function of the inner and outer thermistor positions. For simulations the variables *a* and *b* were stepped. The arrows indicate the directions of increasing values of *a* and *b*.

Figure 12 depicts a contour plot of the simulated peak bridge detuning voltages, *U*_B_(*v*_peak_), representing an efficiency indicator of flow transduction as a function of the thermistors’ positions. There is a flat optimum of this parameter located at the design space border corresponding to the smallest possible values of *b*, *i.e.*, at the inclined border of the contour plot. Small values of *b* lead to a concentration of dissipated power and higher excess temperatures of thermistors due to mutual heating. Higher excess temperatures are preferable for a high bridge detuning signal owing to convective heat transfer. With respect to the membrane center, the best positions of inner and outer thermistors are ≈60 µm and ≈120 µm, respectively. The optimum results from two adverse effects. On the one hand, the excess temperatures are highest if thermistors are grouped near the membrane center. This grouping, on the other hand, approaches Th2 and Th3 and any flow-related temperature reduction of Th2 relative to the temperature of Th3 induces conductive heat transfer from Th3 to Th2. As this heat transfer has the opposite direction of convective heat transfer, it reduces the efficiency of flow transduction.

**Figure 13:**
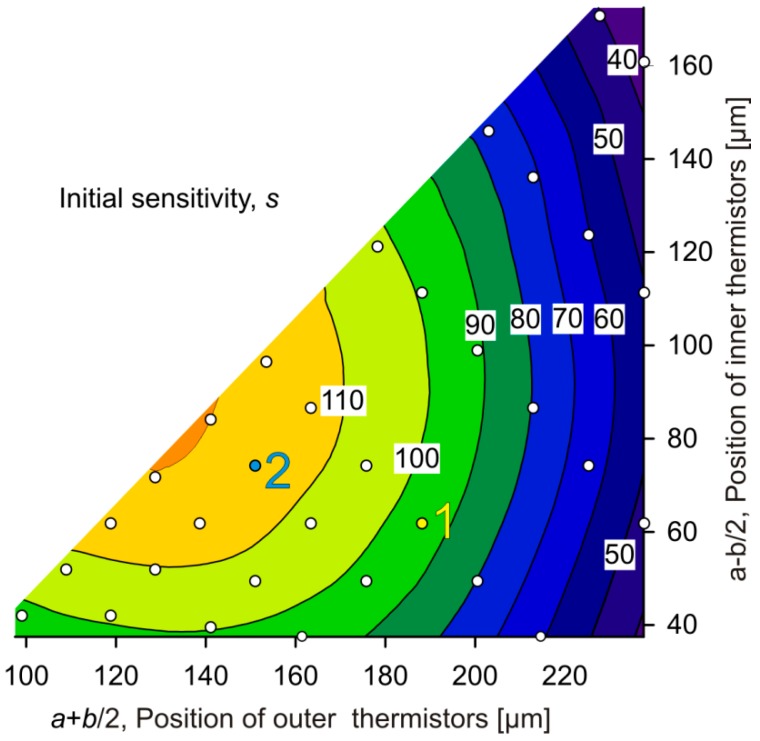
Contour plot of the initial flow sensitivity *s* (mV∙s/m) of the bridge detuning voltage as a function of the thermistor positions.

Figure 13 shows a contour plot derived from simulated data for the initial sensitivity ${s=\partial U_{\text{B}}/\partial v|}_{v=0}$ of the transduction.

The related optimum design resides at the inclined border of the design space, *i.e.*, it appears for minimum values of *b*. The corresponding thermistor positions are ≈135 µm and ≈80 µm measured from the membrane center, or *a* ≈ 110 µm and b ≈ 55 µm. Again the upstream and downstream pairs of thermistors are close together, positioned to efficiently superpose their temperature fields. Obviously, a separation of about 160 µm between Th2 and Th3 is beneficial for high transduction efficiencies in case of a low flow velocity. At low flow velocities, the bridge detuning is mainly due to convective cooling of the upstream thermistors. Tight grouping of the upstream thermistors increases the temperature difference between the fluid and the membrane and enhances flow transduction. With increasing distance between the membrane edge and thermistor pair, the conductive heat transfer to the silicon frame decreases and thus promotes the competing convective heat transfer. As the thermistor pairs are shifted further towards the membrane center, a part of the heat dissipated by the downstream thermistors (mainly Th3) is conducted toward the upstream thermistors, which reduces the bridge detuning voltage. Hence there is an optimum position of the thermistor pairs.

A comparison of Figure 11, Figure 12 and Figure 13 shows that combinations of large measuring range (large *v*_peak_ values) with efficient flow transduction as well as high flow sensitivity may be found only near the lower left corner of the design space. Figure 12 and Figure 13 reveal that a high initial sensitivity is quite compatible with a high maximum bridge detuning voltage. In other words, high initial sensitivity promises a large signal at *v*_max_, too.

**Figure 14:**
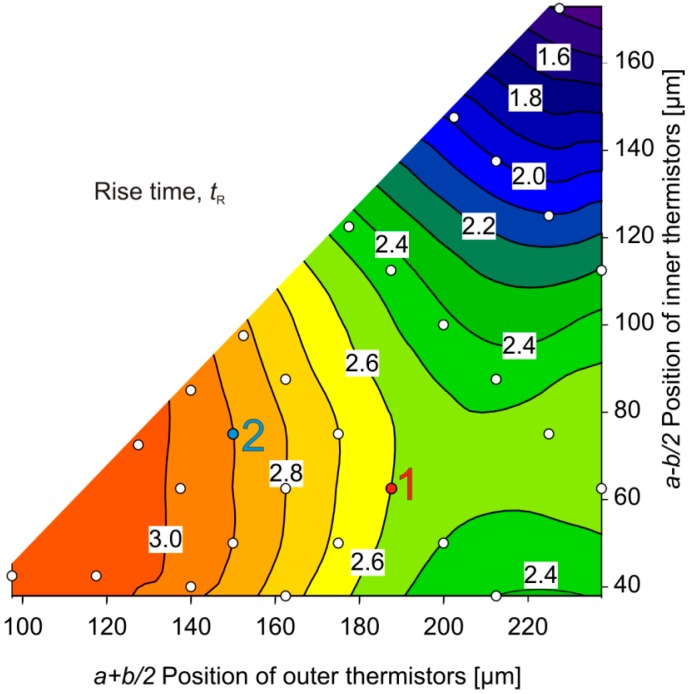
Contour plot of the 10%–90% rise time *t_R_* (ms) as a function of the thermistor positions.

Figure 14 depicts the design dependence of the simulated 10%–90% rise time *t_R_* in response to a step change of the flow velocity from 0 m/s to 1 m/s. For fast responses, all thermistors have to be positioned as close as possible to the membrane boundary, *i.e.*, near the top corner of the design space. From Figure 12 and Figure 13 we see that this requirement excludes highly efficient flow conversion. However, from a comparison of Figure 11 and Figure 14 we see that fast response and a wide region of monotonous flow conversion can be combined easily. The region of fastest response is characterized by a lower thermal resistance between thermistors and the silicon frame acting as heat bath. Then the heat conduction along the membrane as well as via the fluid enables a rapid discharge of the heat capacity of the thermistors.

It was not feasible to verify the data of Figure 14 directly by experiments. Without damage to the membrane, there is no way to induce flow steps at the sensor in the arrangement depicted in Figure 7. However, a response to acoustic shock waves within about 1 ms was found for both designs 1 and 2 using an appropriate setup [23]. This figure is significantly lower than the simulation data of Figure 14, which range about 2.75 µs. Experiments were performed with a flow channel featuring a circular cross-section of about 50 mm radius, whereas the channel of the simulation model measures only 0.5 mm in height. The mismatch can be mainly attributed to a much higher flow velocity gradient that occurs at the transducer surface in the initial phase of shock wave experiments.

## 7. Conclusions

We investigated an advanced thermal flow transduction method, which combines advantages of the calorimetric and the hot-film transduction principle. Four thin-film germanium thermistors were embedded in the silicon nitride membrane and connected to form a Wheatstone bridge. As output quantities, we analyzed both the unbalance voltage across the bridge *U*_B_ utilizing calorimetric action and the total dissipated power *P*_SUP_ applying a constant supply current that represents hot-film anemometric conversion. The calorimetric conversion provides high sensitivity and recognition of the flow direction, whereas the hot-film transduction enables a wide measuring range. Employing highly sensitive thermistors combined with properly designed resistance values, gas-flow transduction requires significantly less than 0.5 mW heating power only, as confirmed by measurements. Evaluating the bridge voltage, an excellent sensitivity of about 94 mV/(m/s) was achieved at low flow velocities utilizing 380 µW of Joule’s heat. The total dissipated power of the bridge exhibits a complementary characteristic that is monotonous at the expense of worse sensitivity and a strong dependence on the fluid temperature. To avoid individual calibration procedures, the resistance of the membrane thermistors must be precisely trimmed. Otherwise, a thorough calibration of the transducer is required for both directions of flow. Moreover, for accurate measurements the fluid and chip temperatures have to be controlled carefully.

Combining bridge detuning and power dissipation characteristics, high-resolution flow measurements are feasible over a large velocity range without ambiguity. This approach avoids sophisticated constant excess temperature transduction operation. The simulation results are in good agreement with corresponding measurement data confirming the basic assumptions and the modeling approach.

Comprehensive FE analyses revealed the main effects of layout variations on sensitivity, conversion efficiency, usable flow range, and rise time. Therefore, a clear design trade-off exists between high initial sensitivity on the one hand and wider measuring range as well as fast response on the other hand.
